# Reduction in potentially inappropriate end-of-life hospital care for cancer patients during the COVID-19 pandemic: A retrospective population-based study

**DOI:** 10.1177/02692163231217373

**Published:** 2023-12-23

**Authors:** Ellis Slotman, Heidi P Fransen, Hanneke WM van Laarhoven, Marieke HJ van den Beuken-van Everdingen, Vivianne CG Tjan-Heijnen, Auke MT Huijben, Agnes Jager, Lia van Zuylen, Evelien JM Kuip, Yvette M van der Linden, Natasja JH Raijmakers, Sabine Siesling

**Affiliations:** 1 Department of Research and Development, Netherlands Comprehensive Cancer Organisation, Utrecht, The Netherlands; 2 Netherlands Association for Palliative Care (PZNL), Utrecht, The Netherlands; 3 Department of Health Technology and Services Research, University of Twente, Technical Medical Centre, Enschede, The Netherlands; 4 Department of Medical Oncology, Amsterdam UMC location University of Amsterdam, Amsterdam, The Netherlands; 5 Cancer Treatment and Quality of Life, Cancer Center Amsterdam, Amsterdam, The Netherlands; 6 Centre of Expertise for Palliative Care, Maastricht University Medical Centre, Maastricht, The Netherlands; 7 Department of Medical Oncology, Maastricht University Medical Centre, Maastricht, The Netherlands; 8 Department of Internal Medicine, Maasstad Hospital, Rotterdam, The Netherlands; 9 Department of Medical Oncology, Erasmus MC Cancer Institute, Erasmus University Medical Center, Rotterdam, The Netherlands; 10 Department of Medical Oncology and Department of Anesthesiology, Pain and Palliative Care, Radboud Medical Center, Nijmegen, The Netherlands; 11 Centre of Expertise in Palliative Care, Leiden University Medical Centre, Leiden, The Netherlands; 12 Department of Radiotherapy, Leiden University Medical Centre, Leiden, The Netherlands

**Keywords:** COVID-19, palliative care, terminal care, neoplasms, cohort studies

## Abstract

**Background:**

The COVID-19 pandemic impacted cancer diagnosis and treatment. However, little is known about end-of-life cancer care during the pandemic.

**Aim:**

To investigate potentially inappropriate end-of-life hospital care for cancer patients before and during the COVID-19 pandemic.

**Design:**

Retrospective population-based cohort study using data from the Netherlands Cancer Registry and the Dutch National Hospital Care Registration. Potentially inappropriate care in the last month of life (chemotherapy administration, >1 emergency room contact, >1 hospitalization, hospitalization >14 days, intensive care unit admission or hospital death) was compared between four COVID-19 periods and corresponding periods in 2018/2019.

**Participants:**

A total of 112,919 cancer patients (⩾18 years) who died between January 2018 and May 2021 were included.

**Results:**

Fewer patients received potentially inappropriate end-of-life care during the COVID-19 pandemic compared to previous years, especially during the first COVID-19 peak (22.4% vs 26.0%). Regression analysis showed lower odds of potentially inappropriate end-of-life care during all COVID-19 periods (between OR 0.81; 95% CI 0.74–0.88 and OR 0.92; 95% CI 0.87–0.97) after adjustment for age, sex and cancer type. For the individual indicators, fewer patients experienced multiple or long hospitalizations, intensive care unit admission or hospital death during the pandemic.

**Conclusions:**

Cancer patients received less potentially inappropriate end-of-life care during the COVID-19 pandemic. Because several factors may have contributed, it is unclear whether this reflects better quality care. However, these findings raise important questions about what pandemic-induced changes in care practices can help provide appropriate end-of-life care for future patients in the context of increasing patient numbers and limited resources.

**What is already known about the topic?**

- Potentially inappropriate end-of-life care in patients with cancer is still common.
- The COVID-19 pandemic has been shown to affect cancer diagnosis and treatment, but evidence on how the pandemic has affected end-of-life care is limited.

**What this paper adds?**

- The COVID-19 pandemic was associated with less potentially inappropriate care at the end of life in patients with cancer.
- The decline in potentially inappropriate end-of-life care was driven by fewer hospitalizations and intensive care unit admissions in the last month of life and fewer hospital deaths.

**Implications for practice, theory or policy**

- The findings of this study raise important questions as to which pandemic related changes in end-of-life care delivery and decision making might be able to contribute to appropriate end-of-life care for future patients.
- Ensuring that awareness for triaging and advance care planning is maintained after the pandemic may be of great importance in this regard.

## Introduction

The COVID-19 pandemic has put an immense burden on healthcare services. The focus shifted towards care for COVID-19 patients, thereby compromising regular care. Previous studies demonstrated the impact of the COVID-19 pandemic on diagnosis and treatment of cancer, including a decline in cancer diagnoses and alterations in diagnostic and treatment pathways.^1–6^

However, less is known about end-of-life care for cancer patients during the COVID-19 pandemic. In the Netherlands, around 45,000 people per year die of cancer.^7^ This means that each year a large group of cancer patients may need end-of-life care. For these patients, the continuation of appropriate end-of-life care during a pandemic is essential. Important elements of appropriate end-of-life care, as described by patients and relatives, are receiving supportive care (e.g. psychological care and symptom control), refraining from intensive life sustaining or life prolonging treatments, receiving care at home and dying at home.^8–10^ There are also aspects of end-of-life care that are seen as potentially inappropriate care, also referred to as aggressive or intensive end-of-life care.^11^ Examples are overuse of chemotherapy, emergency room visits or hospitalizations near death. Potentially inappropriate end-of-life care is still common in patients with cancer^12^ and has been associated with a reduced quality of life of patients and relatives.^9,13,14^

The COVID-19 pandemic led to several changes in care seeking and care delivery that may have impacted care and resource use at the end of life. The pandemic reinforced the importance of palliative care, because of a sharp increase in the number of patients with life-threatening illness.^15–17^ The pandemic also boosted the need for rationing care in the context of limited resources and potential infection risk, thereby increasing awareness for triaging and advance care planning.^18–22^ Timely initiating palliative care and having advance care planning discussions have been associated with reducing the probability of receiving potentially inappropriate end-of-life care.^12,14,23–27^ Additionally, the pandemic and associated restrictive measures potentially caused patients to avoid seeking care, which may also have affected the care received at the end of life.

However, to date little is known about potentially inappropriate end-of-life care in patients dying with cancer during the COVID-19 pandemic. Some studies showed that overall hospital resource use of non-COVID-19 patients declined during the pandemic, including a decline in emergency room visits, hospitalizations and hospital deaths.^28–33^ However, these studies did not specifically focus on the end-of-life phase and only a few reported explicitly on patients with cancer. Therefore, the aim of this study was to investigate potentially inappropriate end-of-life care in patients dying with cancer in the Netherlands before and during the COVID-19 pandemic.

## Materials and methods

### Study design and data

In this retrospective population-based cohort study, linked data of the population-based Netherlands Cancer Registry (NCR) and the Dutch National Hospital Care Registration (LBZ) were used. The NCR is hosted by the Netherlands Comprehensive Cancer Organisation (IKNL) and contains data on diagnosis and treatment of all newly diagnosed malignancies. The LBZ contains nationwide data of patients who received medical care in a Dutch hospital and is hosted by Dutch Hospital Data (DHD). LBZ data about registered care per patient, such as clinical admissions, outpatient contacts and medical procedures, were linked to the NCR.

### Study population

All patients, aged 18 years or older at cancer diagnosis, who died from any cause in the period January 2018 to May 2021 were selected from the NCR-LBZ cohort. Inclusion criteria for this study were a diagnosis of invasive cancer (excluding basal cell or squamous cell skin cancer) or a hospital contact or admission with a registered ICD-10 code for invasive cancer in the year before death (included cancers are shown in Supplemental Table 1). Data were collected on sociodemographic and clinical characteristics (age, sex and cancer type) and on received hospital care. If patients were diagnosed with or received care for multiple cancer types in the year preceding death, the cancer type they were diagnosed with or received care for closest to death was selected.

### Study periods

**Figure 1.**
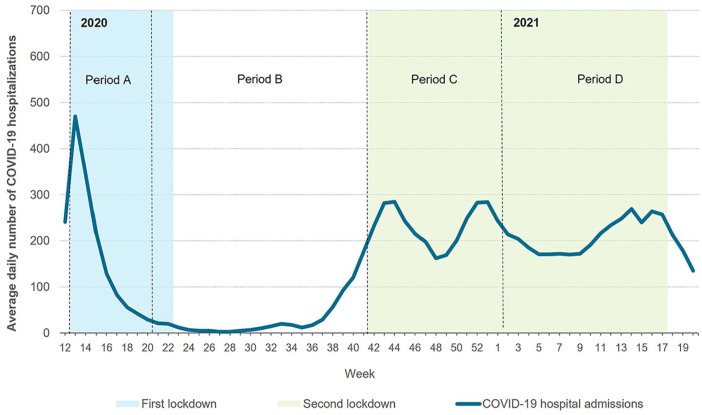
COVID-19 periods based on the number of COVID-19 hospitalizations and the severity of restrictive measures.

For this study, week 12 of 2020 until week 20 of 2021 was considered the total COVID-19 period. This period was divided into four periods based on the number of COVID-19 hospitalizations and the severity of restrictive measures: Period A, weeks 12–20 of 2020 (first peak of COVID-19 hospitalizations and national lockdown); Period B, weeks 21–41 of 2020 (recovery period after first lockdown); Period C, weeks 42–53 of 2020 (second peak of COVID-19 hospitalizations and national lockdown) and Period D, weeks 1–20 of 2021 (prolonged second peak of COVID-19 hospitalizations and extension national lockdown; Figure 1). These same time periods in 2018 and 2019 were considered the reference periods. Patients were assigned to a period based on the start of their end-of-life phase (date of death minus 30 days).

### Outcomes

Potentially inappropriate end-of-life care was assessed based on six frequently used, international, population-based indicators that retrospectively measure potentially inappropriate care in the last month of life: chemotherapy administration, frequency of emergency room (ER) visits (>1), frequency of hospital admissions (>1), frequency of intensive care unit (ICU) admissions (⩾1), length of hospitalizations (>14 days) and hospital death. The selection of these indicators was based on a body of literature concerning the development, validation and benchmarking of these indicators.^11,34–36^ ER visits were included if cancer was the main reason for the visit. Hospital and ICU admissions were included if cancer was either the main reason for the admission or if cancer was present as a secondary diagnosis at the time of the admission. Patients scoring on one or more of the six indicators were defined as having received potentially inappropriate end-of-life care.

### Statistical analysis

Sociodemographic and clinical characteristics of patients were summarized using frequencies and proportions and were compared between the COVID-19 periods and corresponding reference periods using χ^2^ tests. The proportion of patients receiving potentially inappropriate end-of-life care and the proportion of patients scoring on each indicator were compared between the years 2018 and 2019 and the years 2019 and 2020/2021 separately, using χ^2^ tests. Logistic regression analyses were performed to calculate the odds of receiving potentially inappropriate end-of-life care in the COVID-19 periods compared to the corresponding reference periods in 2019, adjusted for differences in age, sex and cancer type. Additionally, the regression analyses were stratified by indicator and age category. Age was categorized into <70 or ⩾70 years, because people aged ⩾70 years had a higher risk of severe COVID-19 infection and therefore stricter protective measures were advised for this group.^37^ Since severe COVID-19 infection in patients with cancer hypothetically impacts the end-of-life care indicators, sensitivity analyses were performed in which patients with cancer who were hospitalized for a COVID-19 infection in their last month of life were excluded. Statistical analyses were performed using Stata version 17.0 software (StataCorp LLC, College Station, Texas, USA). A two-tailed *p*value < 0.01 was considered statistically significant.

## Results

In total, 112,919 patients were included. The majority of the deceased patients were aged ⩾70 years (Table 1). Additionally, in all periods, the proportion of deceased patients with hematological and urological cancers slightly increased over the years, whereas the proportion of deceased patients with gastro-intestinal cancer and lung cancer slightly decreased.

**Table 1.** Sociodemographic and clinical characteristics of all deceased patients by period.

	Period A (weeks 12–20)				Period B (weeks 21–41)			
	2018	2019	First peak	*p*-value	2018	2019	Recovery	*p*-value
	*N* (%)	*N* (%)	2020		*N* (%)	*N* (%)	2020	
			*N* (%)				*N* (%)	
N	5721	5998	6132		13,775	14,083	14,654	
Age (years)				0.11				<0.001^a^
<70	2184 (38)	2204 (37)	2232 (36)		5233 (38)	5130 (36)	5175 (35)	
⩾70	3537 (62)	3794 (63)	3900 (64)		8642 (62)	8953 (64)	9479 (65)	
Sex				0.15				0.23
Male	3246 (57)	3321 (55)	3497 (57)		7808 (57)	7959 (57)	8168 (56)	
Cancer type				0.002^a^				0.003^a^
Breast	393 (7)	436 (7)	454 (7)		912 (7)	932 (7)	1051 (7)	
Gastro-intestinal	1678 (29)	1750 (29)	1721 (28)		3990 (29)	3983 (28)	4033 (28)	
Hematological	421 (7)	466 (8)	532 (9)		1075 (8)	1163 (8)	1241 (9)	
Lung	1433 (25)	1435 (24)	1372 (22)		3496 (25)	3428 (24)	3515 (24)	
Urological	823 (14)	878 (15)	999 (16)		1996 (15)	2107 (15)	2265 (16)	
Other	973 (17)	1033 (17)	1054 (17)		2306 (17)	2470 (18)	2549 (17)	
	Period C (weeks 42-53)				Period D (weeks 1-20)			
	2018	2019	2nd peak	*p*-value	2018	2019	Prolonged 2nd peak	*p*-value
	*N* (%)	*N* (%)	2020		*N* (%)	*N* (%)	2020	
			*N* (%)				*N* (%)	
*N*	7816	8149	8513		13441	13928	13484	
Age (years)				0.001^a^				0.04
<70	2932 (38)	2993 (37)	2958 (35)		5133 (38)	5114 (37)	5026 (37)	
⩾70	4884 (62)	5201 (63)	5555 (65)		8308 (62)	8814 (63)	8458 (63)	
Sex				0.60				0.26
Male	4382 (56)	4631 (56)	4839 (57)		7658 (57)	7807 (56)	7659 (57)	
Cancer type				0.002^a^				<0.001^a^
Breast	543 (7)	614 (8)	667 (8)		966 (7)	970 (7)	1054 (8)	
Gastro-intestinal	2118 (27)	2213 (27)	2229 (26)		3938 (29)	3944 (28)	3672 (27)	
Hematological	660 (8)	688 (8)	750 (9)		1049 (8)	1177 (9)	1207 (9)	
Lung	2016 (26)	2041 (25)	2019 (24)		3320 (25)	3449 (25)	3100 (23)	
Urological	1141 (15)	1424 (15)	1427 (17)		1924 (14)	2084 (15)	2163 (16)	
Other	1338 (17)	1396 (17)	1421 (17)		2244 (17)	2304 (17)	2288 (17)	

a Distribution of categories significantly differs over the years (*p* < 0.01).

### Potentially inappropriate end-of-life care

**Figure 2.**
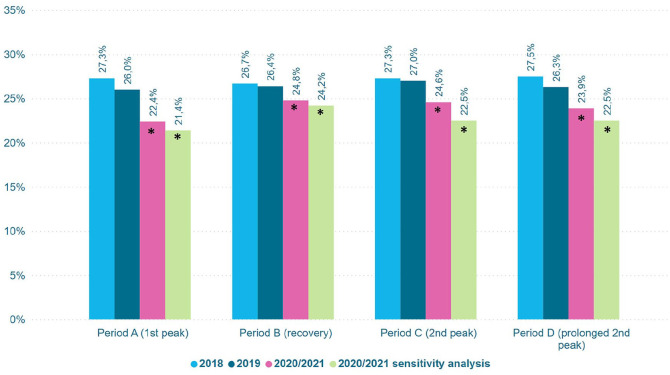
Proportion of patients receiving potentially inappropriate end-of-life hospital care by period. *Significant difference between 2020/2021 and 2019 (*p* < 0.01).

**Figure 3.**
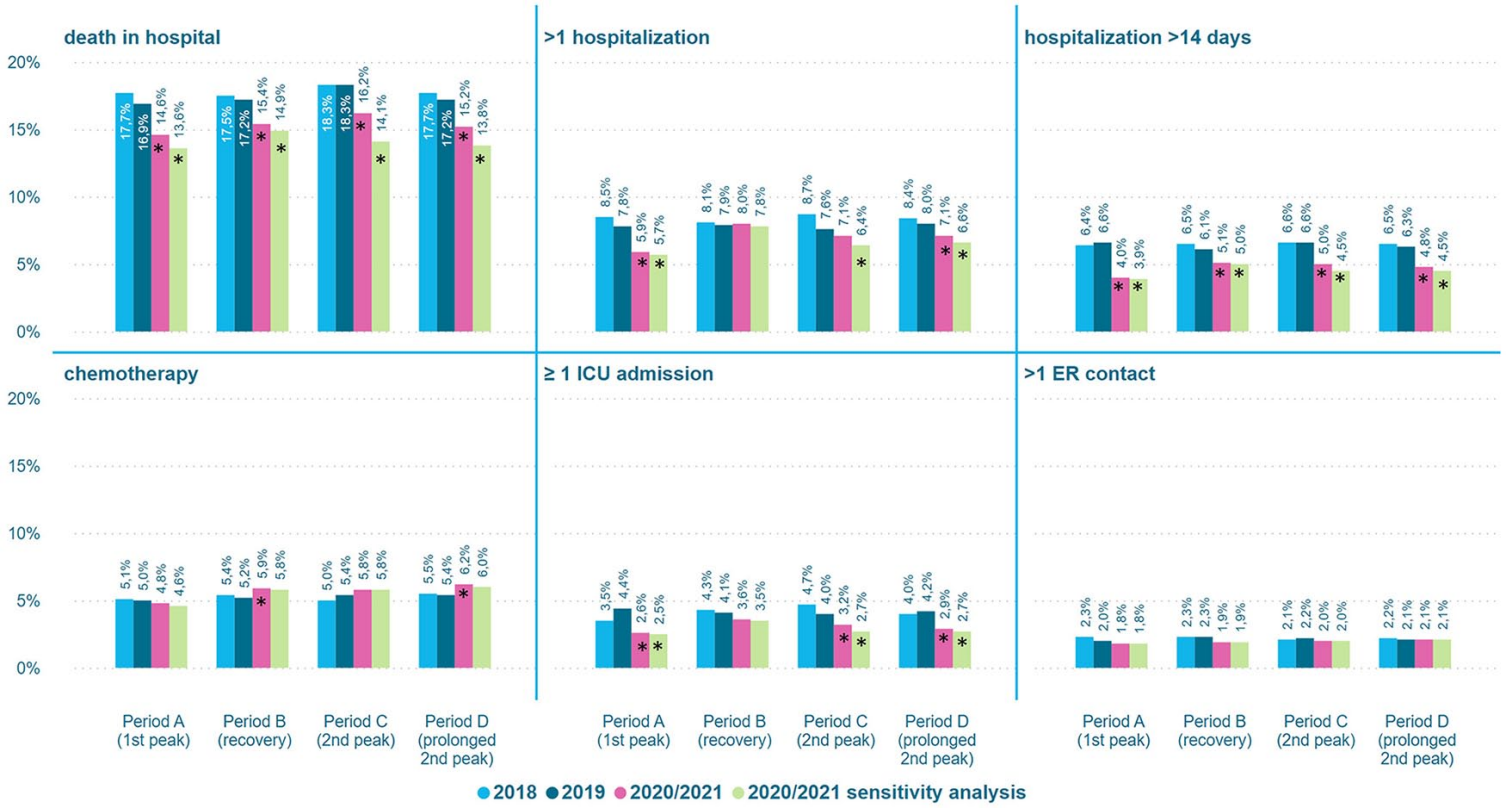
Proportion of patients receiving potentially inappropriate end-of-life hospital care by indicator and period. *Significant difference between 2020/2021 and 2019 (*p* < 0.01).

The proportion of patients receiving potentially inappropriate end-of-life hospital care was significantly lower in all COVID-19 periods compared to the corresponding reference periods, most pronounced during the first peak (period A; Figure 2). In this period the proportion of patients receiving potentially inappropriate end-of-life hospital care decreased from 26.0% in 2019 to 22.4% in 2020 (*p* < 0.001), compared to a decrease from 27.3% to 26.0% between 2018 and 2019. For the individual indicators, the proportion of deaths in hospital, multiple hospitalizations, long hospitalizations and ICU admissions was generally lower in the COVID-19 periods, whereas the proportion of oncological ER contacts remained unchanged (Figure 3). The proportion of patients receiving chemotherapy was higher during the recovery period (period B) and the peak in 2021 (period D). The sensitivity analysis (excluding cancer patients hospitalized for COVID-19 in their last month of life) showed similar results (Figures 2 and 3).

### Multivariable association between the COVID-19 periods and potentially inappropriate end-of-life care

The odds of receiving potentially inappropriate end-of-life hospital care were lower for patients who died during the first peak in 2020 (period A) and the prolonged second peak in 2021 (period D) compared to the reference periods, OR 0.81 (95% CI 0.74–0.88) and OR 0.87 (95% CI 0.82–0.91), respectively (Table 2). For the individual indicators, this holds for ICU admissions, long hospitalizations and hospital deaths. Patients aged ⩾70 years had lower odds of multiple hospitalizations during the first peak (OR 0.68, 95% CI 0.55–0.84).

**Table 2.** Adjusted odds ratios of receiving potentially inappropriate end-of-life hospital care in the COVID-19 periods compared to the corresponding reference periods in 2019.

Indicator	Age group (years)	Period A (first peak)	Period B (recovery period)	Period C (second peak)	Period D (prolonged second peak)
Number of patients	Total	12,130	28,737	16,707	27,412
	<70	4436	10,305	5951	10,140
	⩾70	7694	18,432	10,756	17,272
Odds ratios					
Potentially inappropriate end-of-life care	Total^a^	0.81 (0.74–0.88)^c^	0.92 (0.87–0.97)^c^	0.88 (0.82–0.94)^c^	0.87 (0.82–0.91)^c^
	<70^b^	0.86 (0.75–0.98)^c^	0.93 (0.86–1.01)	0.90 (0.81–1.01)	0.89 (0.82–0.97)^c^
	⩾70^b^	0.78 (0.69–0.87)^c^	0.91 (0.85–0.98)^c^	0.87 (0.79–0.95)^c^	0.86 (0.80–0.92)^c^
Chemotherapy	Total	0.93 (0.78–1.10)	1.17 (1.05–1.29)^c^	1.10 (0.96–1.26)	1.17 (1.05–1.30)^c^
	<70	0.92 (0.74–1.15)	1.22 (1.06–1.40)^c^	1.00 (0.83–1.20)	1.12 (0.97–1.28)
	⩾70	0.96 (0.74–1.23)	1.09 (0.93–1.27)	1.25 (1.02–1.52)	1.26 (1.07–1.47)^c^
>1 ER contact	Total	0.92 (0.70–1.19)	0.82 (0.70–0.97)	0.93 (0.75–1.14)	1.00 (0.85–1.18)
	<70	0.99 (0.69–1.42)	0.93 (0.75–1.17)	0.83 (0.63–1.10)	1.00 (0.80–1.25)
	⩾70	0.84 (0.67–1.24)	0.71 (0.56–0.90)^c^	1.08 (0.79–1.49)	1.01 (0.79–1.29)
>1 hospitalization	Total	0.74 (0.64–0.85)^c^	1.02 (0.94–1.12)	0.94 (0.83–1.05)	0.86 (0.79–0.94)^c^
	<70	0.81 (0.66–0.98)	1.03 (0.92–1.17)	0.86 (0.73–1.01)	0.87 (0.76–0.98)
	⩾70	0.68 (0.55–0.84)^c^	1.02 (0.90–1.15)	1.04 (0.88–1.23)	0.87 (0.76–0.99)
ICU admission	Total	0.56 (0.46–0.69)^c^	0.87 (0.77–0.98)^c^	0.79 (0.67–0.93)^c^	0.66 (0.58–0.75)^c^
	<70	0.47 (0.34–0.64)^c^	0.97 (0.80–1.16)	1.01 (0.79–1.29)	0.64 (0.53–0.78)^c^
	⩾70	0.64 (0.49–0.83)^c^	0.80 (0.68–0.94)^c^	0.64 (0.51–0.81)^c^	0.86 (0.57–0.82)^c^
Hospitalization >14 days	Total	0.59 (0.50–0.69)^c^	0.83 (0.75–0.92)^c^	0.73 (0.64–0.83)^c^	0.74 (0.67–0.82)^c^
	<70	0.63 (0.48–0.82)^c^	0.81 (0.69–0.95)^c^	0.93 (0.75–1.15)	0.72 (0.61–0.85)^c^
	⩾70	0.56 (0.46–0.70)^c^	0.84 (0.74–0.96)^c^	0.63 (0.53–0.74)^c^	0.76 (0.66–0.87)^c^
Hospital death	Total	0.83 (0.75–0.92)^c^	0.88 (0.83–0.94)^c^	0.86 (0.79–0.93)^c^	0.86 (0.80–0.92)^c^
	<70	0.78 (0.67–0.91)^c^	0.86 (0.78–0.95)^c^	0.87 (0.76–0.98)^c^	0.86 (0.78–0.96)^c^
	⩾70	0.87 (0.76–0.98)^c^	0.89 (0.82–0.97)^c^	0.85 (0.77–0.95)^c^	0.86 (0.79–0.94)^c^

ER: emergency room; ICU: intensive care unit.

a Odds ratios and 95% confidence intervals for the total group were adjusted for age, sex and cancer type.

b Odds ratios and 95% confidence intervals for the separate age groups were adjusted for sex and cancer type.

c Significant result (*p* < 0.01).

During the recovery period (period B) and the second peak (period C), patients aged ⩾70 years had lower odds of receiving potentially inappropriate end-of-life hospital care compared to the same periods in 2019, respectively OR 0.91 (95% CI 0.85–0.98) and OR 0.87 (95% CI 0.79–0.95). For the individual indicators, all patients had lower odds of a hospital death during these periods. Patients aged ⩾70 years had lower odds of an ICU admission during both periods, whereas this was not observed for those aged <70 years (*period B*: OR_⩾70_ 0.80 (95% CI 0.68–0.94) versus OR_<70_ 0.97 (95% CI 0.80–1.16); *period C*: OR_⩾70_ 0.64 (95% CI 0.51–0.81) versus OR_<70_ 1.01 (0.79–1.29)). Patients aged <70 years had higher odds of receiving chemotherapy in their last month of life during the recovery period compared to the referen-ce period in 2019 (OR 1.22, 95% CI 1.06–1.40). The sensitivity analysis showed that when excluding patients hospitalized for COVID-19 in the month prior to death, all patients (<70 and ⩾70) had lower odds of receiving potentially inappropriate end-of-life hospital care during the second peak compared to the same period in 2019 (Supplemental Table 2).

## Discussion

### Main findings

Patients with cancer who died during the COVID-19 pandemic received less potentially inappropriate end-of-life hospital care compared to patients dying in the preceding years. This was mainly due to less hospitalizations and ICU admissions in the last month of life and due to fewer patients dying in hospital. Less potentially inappropriate end-of-life care during the COVID-19 pandemic was present in both age groups.

### What this study adds?

The lower rate of potentially inappropriate end-of-life hospital care during the COVID-19 pandemic was driven by less and shorter hospitalizations, less ICU admissions and fewer patients dying in hospital. This might be due to an increased awareness for triaging because of shortness of hospital and ICU bed capacity.^38^ It is likely that for vulnerable patients with cancer, extra care was taken to decide whether hospital admission was sensible and could still be justified in the context of scarce resources. Furthermore, the COVID-19 pandemic prompted the development of a national guideline for advance care planning.^39^ This increased awareness for advance care planning, which has been associated with less potentially inappropriate end-of-life care.^12,14,23–25^ Studies from other countries also showed a substantial increase in documentation of advance directives in several care settings during the pandemic.^20,21^ Patients may have also felt reluctance towards seeking medical care or hospitalization. Visiting restrictions may have been an important factor contributing to this. Healthcare providers and bereaved relatives of patients dying with an without COVID-19 in the Netherlands indicated that visiting restrictions in the last days of life negatively impacted the quality of end-of-life care and the dying process.^40,41^ Additionally, patient reluctance may have been related to fear of COVID-19 infection, which was especially prominent in patients with advanced disease.^42^

While overall fewer patients received potentially inappropriate end-of-life hospital care during the pandemic, chemotherapy administration in the month prior to death slightly increased during the recovery period in 2020 and in the first months of 2021. This may be related to recommendations of the Dutch Society of Medical Oncology to withhold or postpone certain treatments because of capacity restrictions during the COVID-19 peaks, which were lifted as soon as the number of COVID-19 infections declined.^43^ Contributing factors may also have been the evidence that emerged showing no association between chemotherapy administration and COVID-19 mortality,^44^ and a perceived urgency to treat patients during periods with sufficient capacity because of concerns of a possible subsequent COVID-19 peak.

The population based indicators that were used in this study were developed based on the premise that a lower score indicates a situation in which the death of patients is anticipated and measures are taken to prevent aggressive interventions and to provide end-of-life care based on patient preferences.^35^ Therefore, the lower rate of potentially inappropriate end-of-life care during the pandemic would theoretically represent better quality care. During the pandemic awareness for triaging and advance care planning increased. This may have helped patients to achieve end-of-life care according to their preferences, which is often home based care.^8–10^ However, during the COVID-19 pandemic, other aspects may have played a role in the reduction of potentially inappropriate end-of-life care. Patients may have avoided seeking care due to fear of infection and visiting restrictions. Besides this, lack of capacity in hospitals may have prevented patients from receiving care they would have needed or preferred. Therefore, it is unclear whether the lower proportion of patients receiving potentially inappropriate end-of-life hospital care during the pandemic also represents better quality care.

Unfortunately, no systematic data are available about the quality of end-of-life care of patients who died with cancer during the pandemic. A survey study among healthcare providers showed that they were more likely to rate spiritual and emotional end-of-life care as sufficient and perceive the place of death as appropriate when patients died at home compared to when patients died in hospital.^40^ Bereaved relatives of patients who died at home during the pandemic were more often involved in care and treatment decisions and more often perceived the place of death as appropriate compared to bereaved relatives of patients who died in hospital.^41^ More extensive information in this area would be essential in better understanding if the lower rate of potentially inappropriate end-of-life hospital care during the pandemic is more so a reflection of improvements in achieving preference concordant care or of poor quality alternatives because of pressured hospital services.

It is to be expected that the COVID-19 pandemic did not only affect the end-of-life care of cancer patients in the Netherlands. The rapid spread of the pandemic and the large number of COVID-19 patients posed significant challenges to various countries regarding hospital capacity to admit and treat patients with diseases other than COVID-19. In addition, an increased awareness of triaging, advance care planning and palliative care was observed not only in the Netherlands but also in other countries.^15–22^ It is likely that these factors have influenced end-of-life care practices and the rate of potentially inappropriate end-of-life care in other countries in a manner similar to that presented in our study.

As we exit the pandemic, the challenge of providing appropriate end-of-life care to an increasing number of patients in the context of restricted resources and capacity will stay equally relevant. It is expected that the number of patients dying of cancer will increase with 17% between 2019 and 2032 (46,000–54,000).^45^ Shortages of medical staff are expected to more than double during this period.^46^ These challenges are not unique to the Dutch healthcare setting.^47,48^ The results of this study can contribute to a critical evaluation of which changes in end-of-life care practices have contributed to a reduction in potentially inappropriate care, and which of these changes could help to provide future patients with end-of-life care that is of added value while at the same time using resources efficiently. For example, maintaining the awareness for advance care planning and triaging is of great importance since it can assist in providing preference concordant end-of-life care while limiting potentially inappropriate, non-beneficial and expensive resource use at the end of life.

### Strengths and limitations of the study

A main strength of this study is the use of data from a linkage between two population-based registries, thereby being able to report on potentially inappropriate end-of-life hospital care on a nationwide scale. Besides this, different periods of intensity of the pandemic were studied. However, the results should be interpreted in the light of some limitations. First, this study was conducted using administrative health care data, which is not primarily collected for the purpose of research. Therefore, no detailed information was available on the content of the care that was provided nor on other possible determinants of potentially inappropriate end-of-life care, such as patient preferences and use of palliative and supportive care services. Second, it could not be determined if a patient actually died of cancer, since no cause of death information was available. Although efforts have been made to make the best possible selection of patients who likely died of advanced cancer, patients that died (unexpectedly) from other causes may still have been included. For these patients, their chosen end-of-life care may have been appropriate, as their expected prognosis may have been good or unknown. Third, this study used indicators that indicate the appropriateness of end-of-life care at the population level, but not the appropriateness of care for an individual. Clinical factors may justify interventions at the end of life and patient preferences vary. Additionally, due to the retrospective design this study did not include patients who survived and may have benefited from the received interventions. For these reasons, the estimates of potentially inappropriate end-of-life care should be interpreted with caution.

## Conclusion

This study showed a significant reduction of potentially inappropriate end-of-life hospital care for cancer patients during the COVID-19 pandemic, not just during the pandemic peaks but between these peaks as well. This was driven by less hospital and ICU admissions and less deaths in hospital, indicating that end-of-life care for patients with cancer was provided more often at home or in home-like settings. Lack of capacity in hospitals, reluctance of patients to go to the hospital and an increased awareness for triaging and advance care planning may all have been contributing factors to the reduction in potentially inappropriate end-of-life hospital care. Therefore, it is unclear whether this represents better quality care. Further research could investigate the underlying reasons for the reduction in potentially inappropriate end-of-life care and whether the declining trend persisted after the pandemic.

## Supplemental Material

sj-pdf-1-pmj-10.1177_02692163231217373 – Supplemental material for Reduction in potentially inappropriate end-of-life hospital care for cancer patients during the COVID-19 pandemic: A retrospective population-based study Click here for additional data file. Supplemental material, sj-pdf-1-pmj-10.1177_02692163231217373 for Reduction in potentially inappropriate end-of-life hospital care for cancer patients during the COVID-19 pandemic: A retrospective population-based study by Ellis Slotman, Heidi P Fransen, Hanneke WM van Laarhoven, Marieke HJ van den Beuken-van Everdingen, Vivianne CG Tjan-Heijnen, Auke MT Huijben, Agnes Jager, Lia van Zuylen, Evelien JM Kuip, Yvette M van der Linden, Natasja JH Raijmakers and Sabine Siesling in Palliative Medicine
