# Supplementary material for: Reduction in potentially inappropriate end-of-life hospital care for cancer patients during the COVID-19 pandemic: A retrospective population-based study
Source: Palliat Med. 2023 Dec 23;38(1):140–9. doi: 10.1177/02692163231217373 (PMC10798006; doi:10.1177/02692163231217373)
Supplement: sj-pdf-1-pmj-10.1177_02692163231217373 – Supplemental material for Reduction in potentially inappropriate end-of-life hospital care for cancer patients during the COVID-19 pandemic: A retrospective population-based study [file sj-pdf-1-pmj-10.1177_02692163231217373.pdf]

Supplemental Material

Supplemental Table 1: Included cancers and their categorization based on the International Classification of Diseases 10th Revision (ICD-10) 2

Supplemental Table 2: Adjusted odds ratios of receiving potentially inappropriate care in the COVID-19 periods compared to the corresponding reference periods in 2019 (sensitivity analysis) 3

Supplemental Table 1: Included cancers and their categorization based on the International Classification of Diseases 10th Revision (ICD-10)

**Cancer type**	**Subgroups**	**ICD-10 codes**
Breast cancer	Invasive breast cancer	C50
Gastro-intestinal cancer	Esophageal cancer	C15
	Stomach cancer	C16
	Cancer of the small intestine	C17
	Colon cancer	C18
	Cancer of the rectosigmoid junction	C19
	Rectum cancer	C20
	Cancer of the liver and intrahepatic bile ducts	C22
	Pancreatic cancer	C25
Hematological cancer	Lymphomas	C81-C86
	Malignant immunoproliferative diseases	C88
	Multiple myeloma and malignant plasma cell tumors	C90
	Leukemias	C91-C95
	Other and unspecified hematological malignancies	C96
Lung cancer	Cancer of the trachea	C33
	Cancer of the bronchus and lung	C34
Urological cancer	Prostate cancer	C61
	Kidney cancer	C64,C65
	Cancer of the ureter	C66
	Bladder cancer	C67
Other	Cancer of the brain and central nervous system	C70-C72
	Melanoma of the skin	C43
	Cancer of the lip, oral cavity and pharynx	C00-C14
	Cancer of the nasal cavity and middle ear	C30
	Cancer of the larynx	C32
	Cervical cancer	C53
	Ovarian cancer	C56

Supplemental Table 2: Adjusted odds ratios of receiving potentially inappropriate care in the COVID-19 periods compared to the corresponding reference periods in 2019 (sensitivity analysis)

**Indicator**	**Age group**	**Period A (1st peak)**	**Period B (recovery period)**	**Period C (2nd peak)**	**Period D (prolonged 2nd peak)**
Number of patients	Total	12037	28617	16440	27135
	<70	4412	10269	5870	10049
	≥70	7625	18348	10570	17086
Potentially inappropriate EOL care	Total [a]	0.76 (0.70-0.83)[c]	0.90 (0.85-0.95)[c]	0.79 (0.73-0.85)[c]	0.81 (0.76-0.85)[c]
	<70[b]	0.84 (0.73-0.95)[c]	0.92 (0.84-1.00)	0.84 (0.75-0.94)[c]	0.84 (0.77-0.92)[c]
	≥70[b]	0.72 (0.65-0.81)[c]	0.88 (0.82-0.95)[c]	0.76 (0.69-0.84)[c]	0.78 (0.73-0.84)[c]
Chemotherapy	Total	0.90 (0.76-1.06)	1.15 (1.04-1.27)[c]	1.11 (0.97-1.27)	1.13 (1.02-1.25)
	<70	0.91 (0.73-1.14)	1.20 (1.05-1.38)[c]	1.00 (0.83-1.21)	1.08 (0.94-1.24)
	≥70	0.90 (0.69-1.16)	1.08 (0.92-1.26)	1.25 (1.02-1.52)	1.20 (1.03-1.41)
>1 ER contact	Total	0.92 (0.70-1.20)	0.82 (0.69-0.96)	0.93 (0.75-1.15)	1.01 (0.86-1.19)
	<70	1.00 (0.70-1.44)	0.93 (0.74-1.16)	0.84 (0.63-1.11)	1.01 (0.81-1.26)
	≥70	0.84 (0.57-1.24)	0.71 (0.56-0.90)[c]	1.08 (0.79-1.50)	1.02 (0.79-1.30)
>1 hospitalization	Total	0.71 (0.61-0.82)[c]	1.00 (0.92-1.09)	0.85 (0.75-0.96)[c]	0.80 (0.73-0.88)[c]
	<70	0.79 (0.65-0.97)	1.02 (0.90-1.15)	0.79 (0.67-0.94)[c]	0.83 (0.73-0.94)[c]
	≥70	0.63 (0.51-0.78)[c]	0.98 (0.87-1.11)	0.93 (0.78-1.10)	0.79 (0.69-0.91)[c]
ICU admission	Total	0.54 (0.44-0.66)[c]	0.85 (0.75-0.95)[c]	0.65 (0.55-0.78)[c]	0.61 (0.54-0.70)[c]
	<70	0.44 (0.32-0.61)[c]	0.94 (0.78-1.13)	0.88 (0.68-1.13)	0.60 (0.49-0.73)[c]
	≥70	0.61 (0.46-0.80)[c]	0.78 (0.66-0.92)[c]	0.51 (0.40-0.65)[c]	0.64 (0.53-0.77)[c]
Hospitalization >14 days	Total	0.57 (0.48-0.67)[c]	0.81 (0.74-0.90)[c]	0.66 (0.58-0.76)[c]	0.69 (0.62-0.77)[c]
	<70	0.61 (0.47-0.79)[c]	0.80 (0.68-0.94)[c]	0.85 (0.68-1.06)	0.68 (0.57-0.80)[c]
	≥70	0.54 (0.44-0.67)[c]	0.83 (0.72-0.94)[c]	0.57 (0.48-0.68)[c]	0.71 (0.62-0.82)[c]
Hospital death	Total	0.77 (0.69-0.85)[c]	0.84 (0.79-0.90)[c]	0.73 (0.67-0.80)[c]	0.77 (0.72-0.82)[c]
	<70	0.75 (0.64-0.87)[c]	0.84 (0.76-0.93)[c]	0.78 (0.68-0.90)[c]	0.80 (0.72-0.88)[c]
	≥70	0.79 (0.70-0.90)[c]	0.85 (0.78-0.92)[c]	0.71 (0.63-0.79)[c]	0.76 (0.69-0.82)[c]

Abbreviations: EOL = end of life, ER = emergency room, ICU = intensive care unit

[a]Odds ratios and 95% confidence intervals for the total group were adjusted for age, sex and cancer type.

[b]Odds ratios and 95% confidence intervals for the separate age groups were adjusted for sex and cancer type.

[c]Significant result (P<0.01).
